# Reprogramming prostate cancer through the microbiome

**DOI:** 10.3389/fmed.2025.1690498

**Published:** 2025-11-26

**Authors:** Jhommara Bautista, Walter D. Cardona-Maya, Kelly Gancino-Guevara, Andrés López-Cortés

**Affiliations:** 1 Cancer Research Group (CRG), Faculty of Medicine, Universidad de Las Américas, Quito, Ecuador; 2 Grupo Reproducción, Departamento de Microbiología y Parasitología, Facultad de Medicina, Universidad de Antioquia, Medellín, Colombia

**Keywords:** prostate cancer, dysbiosis, probiotics, fecal microbiota transplantation, microbiome-based therapeutic approaches

## Abstract

Prostate cancer (PCa) is a major global public health challenge, driven by a multifactorial interplay of genetic, epigenetic, hormonal and environmental determinants. In recent years, the human microbiome has emerged as a critical and previously underappreciated contributor to PCa initiation, progression, and therapeutic response. Emerging high-resolution multi-omics studies have demonstrated that microbial communities across the gut, urinary tract and prostate form a functional axis that shapes immune surveillance, hormonal metabolism, inflammatory tone and epigenetic regulation. Dysbiosis in these compartments promotes chronic inflammation, modulates androgen receptor signaling, and produces bioactive metabolites, including short-chain fatty acids, that activate oncogenic IGF-1/MAPK/PI3K and NF-κB/JAK/STAT pathways. Cross-compartmental trafficking of bacterial taxa and metabolites reinforces tumor-promoting circuits, while specific commensals such as *Akkermansia muciniphila* enhance antitumor immunity and improve responses to androgen deprivation therapy. Importantly, microbiota-derived factors also modulate microRNA (miRNAs) expression and epigenetic signatures, thereby affecting tumor plasticity and resistance to therapy. These mechanistic insights have catalyzed interest in microbiome-based therapeutic approaches, including probiotics, prebiotics, fecal microbiota transplantation, dietary modulation and bacteriophage therapy, which hold promise for restoring eubiosis and enhancing treatment efficacy. Nevertheless, clinical translation remains limited by inter-individual variability and the need for well-designed, longitudinal studies integrating shotgun metagenomics, metabolomics and host-microbe interactomics. Overall, the prostate, urinary and gut microbiomes represent interconnected targets that may inform precision diagnostics and novel therapeutic strategies in PCa.

## Introduction

Prostate cancer (PCa) remains a major global health challenge, representing approximately 7% of all cancers in men and ranking as the third most common non-cutaneous malignancy worldwide (1, 2). Incidence varies markedly across regions, from 6.3 to 83.4 cases per 100,000 individuals, with the highest rates reported in Northern Europe, sub-Saharan Africa, and the Caribbean, and the lowest in parts of Central and South Asia (3, 4). Mortality is substantial: PCa is the fifth leading cause of cancer-related deaths among men, responsible for roughly 23% of deaths in those diagnosed (5, 6). Survivors frequently endure chronic complications, including urinary, sexual, and reproductive dysfunction, that profoundly affect quality of life. While disparities in screening, healthcare access, and diagnostic infrastructure contribute to the geographical heterogeneity, PCa incidence and aggressiveness reflect a multifactorial interplay among genetic, epigenetic, hormonal, environmental, and lifestyle determinants (7, 8).

Recent multi-omic studies have provided compelling evidence that prostate carcinogenesis is influenced not only by genetic and hormonal determinants but also by host-microbiome-immune interactions that modulate systemic metabolic and inflammatory tone. Integrative analyses combining metagenomics, transcriptomics, and metabolomics reveal that dysbiosis in gut and urogenital microbiota alters lipid and amino-acid metabolism, fostering a pro-oncogenic milieu characterized by chronic inflammation, oxidative stress, and androgen receptor hyperactivation. In particular, enrichment of *Bacteroides*, *Ruminococcus*, and *Akkermansia* species has been linked to enhanced production of short-chain fatty acids and bile acid derivatives that sustain insulin-like growth factor-1 (IGF-1) and PI3K/AKT signaling, promoting epithelial proliferation and immune evasion. Concomitantly, depletion of anti-inflammatory taxa such as *Faecalibacterium* and *Bifidobacterium* reduces regulatory T-cell function and impairs mucosal barrier integrity, further amplifying systemic inflammation and facilitating tumor progression (9–11).

Beyond compositional changes, these studies underscore functional consequences of microbial reprogramming in prostate carcinogenesis: upregulation of genes involved in steroid metabolism, increased expression of pro-inflammatory cytokines (IL-6, TNF-*α*), and metabolic shifts toward aerobic glycolysis within the tumor microenvironment. Critically, this microbiome-driven biology intersects with established non-genetic risk factors—age, ethnicity, diet, obesity, tobacco exposure, early sexual debut, sexually transmitted infections, and chronic prostatic inflammation—by modulating endocrine and immune axes central to disease progression (12–14).

In addition to these classical determinants, the human microbiome has emerged as a pivotal and dynamic regulator of prostate tumor biology. Microbial communities residing across the gut, urinary tract, and prostate exert profound effects on local and systemic immunity, hormonal metabolism, and inflammatory tone. Dysbiosis within these niches contributes to oncogenic transformation by promoting oxidative stress, chronic inflammation, and aberrant androgen receptor (AR) signaling (12–14). Previous reviews primarily established foundational mechanistic links between microbial dysbiosis and PCa pathogenesis. Building on these earlier frameworks, the present work advances the field by integrating high-resolution multi-omic, immunometabolic, and translational evidence published through 2025. This synthesis moves beyond gut-centric perspectives to conceptualize a functional gut-urinary-prostate axis, where cross-compartment microbial trafficking, metabolite exchange, and endocrine-immune crosstalk converge to influence tumor initiation, progression, and therapeutic response. By unifying recent metagenomic, metabolomic, and host-interactome findings, this review provides a more comprehensive and dynamic model of how microbial ecosystems shape prostate tumor biology (15).

Multi-cohort evidence refines how dysbiosis interfaces with prostate carcinogenesis: gut-driven inflammation and microbially derived metabolites act as systemic modulators of androgen-dependent tumor biology, engaging pattern-recognition pathways that converge on NF-κB/STAT3 signaling and sustain a pro-neoplastic immune-metabolic state. Loss of barrier-protective commensals increases permeability and endotoxemia, amplifying IL-6/TNF-*α* tone, while microbially mediated steroid conversions can maintain AR activity despite androgen-deprivation (16, 17). Diet further exacerbates this axis, Western/high-fat patterns enrich bile acid-transforming microbes that intensify oxidative stress and DNA damage, together positioning the gut-prostate conduit as a mechanistically actionable target in PCa (15, 18, 19).

Emerging evidence links circadian regulation to microbiome-mediated oncogenic pathways, introducing a temporal layer to prostate cancer biology. The circadian system governs endocrine, immune, and metabolic balance through molecular clocks in central and peripheral tissues, including the gut-prostate axis. Disruption of this synchrony by irregular feeding or metabolic stress induces dysbiosis marked by altered rhythmicity of *Bacteroides, Lactobacillus*, and *Clostridiales* and reduced production of short-chain fatty acids and bile acid derivatives. These metabolites modulate host clock genes (BMAL1, PER1/2), oxidative stress, and nuclear receptor pathways (PPARγ, FXR), influencing androgen receptor activity and tumor metabolism (R10). In PCa, circadian misalignment and dysbiosis synergize to promote inflammation, epigenetic reprogramming, and therapeutic resistance. Conversely, chrononutritional strategies, such as time-restricted feeding and melatonin-based interventions, may restore rhythmic microbial oscillations and metabolic homeostasis (20, 21).

Recent evidence further underscores the clinical and molecular relevance of microbiome alterations in prostate cancer. Multi-omics profiling has revealed that gut microbial signatures are intricately linked with androgen metabolism, immune reprogramming, and metabolic homeostasis in prostate carcinogenesis. Functional enrichment analyses from metagenomic datasets demonstrate that taxa enriched in PCa patients exhibit upregulated pathways for steroid biosynthesis, fatty acid metabolism, and nucleotide repair, collectively sustaining proliferative and inflammatory phenotypes. Moreover, distinct microbial configurations have been associated with differential expression of androgen receptor-regulated transcripts and tumor immune infiltration patterns, suggesting that the microbiome exerts dual control over hormonal and immune axes. Longitudinal analyses indicate that dysbiosis precedes biochemical recurrence and can predict therapy response, particularly to androgen deprivation therapy (ADT) and immune checkpoint blockade, emphasizing its prognostic value (22–24).

In recent years, an additional dimension has emerged: many of these risk factors directly influence the composition, diversity, and metabolic activity of the human microbiota—a complex, host-associated network of bacteria, fungi, viruses, and archaea that shapes immune surveillance, hormonal metabolism, and inflammatory tone (25–27). High-resolution multi-omic techniques have revealed that microbial communities are not restricted to the gut but also inhabit the prostate, urinary tract and other urogenital niches, exerting both local and systemic effects on host physiology. Dysbiosis, defined as a sustained disruption of microbial homeostasis, has been associated with a growing list of pathologies including metabolic diseases, immune-mediated disorders and malignancies (11, 28). In cancer, these effects are mediated through the tumor microenvironment (TME), a dynamic cellular and molecular ecosystem in which microbes interact with malignant cells, immune infiltrates and stromal components to shape tumor initiation, progression and therapy response.

Specific to PCa, diverse microbial taxa have been implicated in carcinogenesis and tumor progression. Pathogens such as *Escherichia coli*, *Fusobacterium nucleatum* and Cutibacterium acnes have been associated with chronic inflammation, oxidative stress and DNA damage, whereas other genera (e.g., *Faecalibacterium prausnitzii, Corynebacterium*) influence local immune tone, metabolite production and biofilm formation. These associations, however, are not always consistent across studies; divergent findings may reflect differences in sequencing technique (16S vs. shotgun metagenomics), cohort characteristics, dietary habits or prior treatment exposure (29–31). A mounting body of mechanistic research positions the microbiome not as a passive correlate of disease, but as an active, modulatory axis in PCa biology.

Several converging lines of evidence support this paradigm shift. Gut microbial communities modulate tumor growth, treatment efficacy and resistance in both hormone-sensitive and castration-resistant PCa. Dysbiosis enriched in short-chain fatty acid (SCFA)-producing taxa such as *Ruminococcus*, *Alistipes* and *Phascolarctobacterium* accelerates tumor progression through SCFA-mediated activation of IGF-1/MAPK/PI3K signaling, induction of autophagy and polarization of macrophages toward a tumor-promoting M2 phenotype (32, 33). Beyond metabolic regulation, certain gut bacteria directly alter systemic androgen availability; taxa capable of converting androgen precursors into active testosterone and dihydrotestosterone sustain tumor growth under androgen deprivation therapy (ADT), subsequently undermining one of the principal approaches used for advanced PCa (17). Preclinical models further support microbiota-tumor crosstalk; fecal microbiota transplantation from patients with high tumor burden accelerates disease progression in germ-free and TRAMP mice, which is strongly influenced by dietary composition and associated shifts in microbial metabolism (16). Conversely, certain commensals, such as *Akkermansia muciniphila*, enhance anti-tumor immunity and improve ADT responsiveness, illustrating that microbiota composition can be harnessed for therapeutic benefit (34). Nonetheless, these mechanistic findings are largely derived from preclinical studies, and causal effects in humans remain to be demonstrated in appropriately designed interventional trials.

Recent multi-cohort metagenomic and GWAS analyses reveal population-specific microbial signatures associated with PCa aggressiveness, while integrative metabolomic data highlight how gut-derived metabolites modulate AR signaling and DNA-damage repair pathways. Furthermore, dietary and obesity-related perturbations of the microbiota are now recognized as key modifiers of prostatic inflammation and immune evasion. Collectively, these findings indicate that microbiota-host interactions operate at multiple biological layers, metabolic, endocrine, and immunologic, creating a fertile ground for precision interventions (16, 35, 36).

Critically, the relevant microbiome in PCa is not limited to the gut ecosystem. The urinary tract contains resident microbial communities whose proximity to the prostate positions them as potential modulators of local inflammation, immune tone and tumor behavior. Alterations in urinary microbiota have been associated with PCa presence, tumor grade and specific inflammatory profiles, and may act as non-invasive biomarkers for diagnosis or risk stratification (16, 25, 26). Considering the anatomical and functional continuity between gut, urinary tract and prostate, microbial metabolites or whole bacteria may traffic across compartments, integrating local dysbiosis into systemic tumor-promoting networks (26). This concept supports the emerging view of a gut-urinary-prostate axis, in which distinct but interconnected microbial ecosystems collectively contribute to malignant transformation and progression.

This evolving perspective demands a shift in research and clinical focus—from cataloging compositional differences to elucidating mechanistic pathways and identifying actionable microbial targets. While 16S rRNA sequencing has been instrumental in profiling microbiota structure, the field is rapidly moving toward shotgun metagenomics, metatranscriptomics, metabolomics and host-microbe interactomics to uncover functional capabilities and causal relationships. These approaches have already revealed microbial signatures associated with high-grade PCa, therapy resistance and activation of specific oncogenic signaling cascades (16, 32, 37). They also highlight the bidirectional nature of microbiome-therapy interactions, in which systemic therapies reshape microbial communities and the altered microbiota in turn modulates drug metabolism, immune activation and endocrine balance.

Recent studies have emphasized the concept of the *oncomicrobiome*, describing how specific microbial communities actively contribute to tumor evolution and therapeutic resistance in PCa. In particular, dysbiotic microbial profiles have been associated with reduced efficacy of androgen deprivation therapy and immune checkpoint inhibitors, suggesting that microbial metabolites and immune-modulatory signals may promote resistance mechanisms (38, 39). Moreover, emerging evidence indicates that the prostate microbiome may influence tumor biology at the epigenetic level by modulating miRNAs expression patterns involved in cellular proliferation, apoptosis and immune escape. For example, microbiota-derived metabolites have been shown to regulate oncogenic miRNAs and DNA methylation signatures, contributing to a more aggressive tumor phenotype (40, 41).

Accordingly, this review delivers an updated and integrative synthesis of the mechanistic and translational landscape linking the microbiome to PCa. It delineates the compositional and functional attributes of the gut, urinary, and prostatic microbiota associated with tumor initiation, progression, and therapeutic response, while elucidating the molecular and immunometabolic pathways through which microbial metabolites, inflammatory signaling, and androgen receptor (AR) modulation converge to shape disease biology. By integrating preclinical and clinical evidence, we emphasize the microbiome as an active etiopathogenic driver rather than a passive correlate of PCa, and explore its translational potential in diagnostics and therapy. Particular attention is given to emerging microbiome-based strategies, including probiotics, dietary modulation, engineered microbial consortia, and fecal microbiota transplantation (FMT), that exemplify precision approaches for reprogramming the tumor microenvironment and improving treatment outcomes.

## Microbiome and its relationship to prostate cancer

### Microbiome composition in prostate cancer

Recent research has revealed a robust association between the composition of the prostatic microbiome and PCa, indicating that microbial influences extend beyond passive colonization to active modulation of tumor biology (42). The prostate harbors a diverse and anatomically specialized microbial ecosystem comprising bacteria, fungi, viruses, and parasites, which can interact with local tissues to regulate immune responses, inflammation, and metabolic activity (26, 43). Advances in 16S rRNA sequencing and, more recently, multi-omics approaches have enabled finer taxonomic and functional resolution of this ecosystem (30, 44). Genera such as *Streptococcus*, *Lactobacillus*, *Vibrio*, *Campylobacter*, and *Propionibacterium* have been detected, each with context-dependent roles in inflammation, immune modulation, and metabolite production (26, 45). This highlights that functional outputs (e.g., metabolite production, inflammatory triggering and biofilm formation) are often more relevant than mere taxonomic presence in the context of tumor-promoting processes.

The composition of the prostatic microbiota is shaped by multiple determinants, including host genetics, diet, physical activity, sexual history, and interactions with neighboring microbial niches, most notably the gut, urinary tract, and seminal fluid. Epidemiological and experimental evidence supports a bidirectional relationship between the gut and prostatic microbiota, commonly referred to as the *enteroprostatic axis*, mediated in part by diet-dependent systemic metabolite fluxes. For example, the insulin-like IGF-1 axis, a well-established driver of PCa cell proliferation and survival, can be modulated by microbial metabolites originating in the gut (46–48). Murine models have further shown that gut microbiota regulate epithelial permeability, hormone metabolism, and the inflammatory status of the prostate (47). Microbial overlap between prostate, urine, and semen also suggests potential ascending infection routes along the urogenital tract (45), with *Corynebacterium*, *Staphylococcus*, and *Lactobacillus* among the dominant genera. Of note, lactic acid-producing bacteria in the urinary tract may inhibit pathogen colonization, offering a protective effect on prostatic health (29, 49). Collectively, these interactions support the emerging concept of an *enteroprostatic axis*, in which distinct but connected microbial niches coordinate metabolic and inflammatory signals relevant to tumor biology.

In parallel, the tumor microbiome in PCa exhibits distinct compositional and functional features compared to healthy prostate tissue. Microorganisms may reside locally within the gland or translocate from distant reservoirs such as the gut or urinary tract (50, 51). Bioinformatics-driven comparative analyses consistently demonstrate significant taxonomic shifts, microbial dysbiosis, that correlate with tumor presence, grade, and biological aggressiveness (27, 44). Mechanistically, dysbiosis is thought to promote malignancy through persistent inflammation and reprogramming of host metabolic pathways (43). Predominant taxa in tumor tissue include *Pseudomonas, Actinobacteria, Streptococcus, Staphylococcus, Corynebacterium, Mycobacterium,* and *Moraxella* (44, 52). Among these, *Cutibacterium acnes* is of particular interest for its ability to sustain oxidative stress-driven inflammation, potentially inducing mutagenic DNA damage or epigenetic alterations that promote tumor development (53). Interestingly, while several studies report enrichment of genera such as *Lachnospira* and *Akkermansia* in aggressive PCa, other cohorts show an opposite trend or no association, highlighting inter-cohort variability and the need for a more standardized analytical framework (31, 44).

Quantitative differences in microbial composition also appear to align with disease severity. High-grade tumors frequently show enrichment of *Cutibacterium* and *Corynebacterium*, suggesting that certain microbial signatures may serve as biomarkers for aggressive disease phenotypes (54, 55). Conversely, reductions in potentially protective commensals such as *Streptococcus* and *Lactobacillus* may impair metabolic homeostasis and anti-inflammatory capacity, facilitating a tumor-promoting milieu (47). Bacterial metabolites further amplify oncogenic processes; for example, lipopolysaccharides (LPS) activate NF-κB signaling, enhancing cancer cell migration, proliferation, and inflammatory cytokine production. Other genera, such as *Corynebacterium*, display biofilm-forming and invasive traits, while diet-associated enrichment of *Propionibacterium*, *Staphylococcus*, and *Ochrobactrum* in tumor-associated tissue has been observed, linking dietary patterns to microbiota-driven tumor modulation. Therefore, profiling only taxonomic abundance may be insufficient to understand the oncogenic potential of individual taxa unless functional outputs are concurrently evaluated.

### Microbiota changes in prostate tumors

The tumor microbiome is composed of complex microbial communities that can influence tumor immunity, modulate immune cell activity, and shape therapeutic outcomes. Microorganisms may be present locally within the prostate or may translocate from distant reservoirs such as the gut and urinary tract Bioinformatics-driven analyses have consistently shown significant differences in microbial composition between healthy and cancerous prostate tissue, pointing to microbial dysbiosis as a contributor to cancer initiation and progression chronic inflammation and altered metabolic pathways are two key mechanisms by which dysbiosis may promote malignancy predominant taxa in tumor tissue include *Pseudomonas*, *Actinobacteria*, *Streptococcus*, *Staphylococcus*, *Corynebacterium*, *Mycobacterium*, and *Moraxella* Among these, *Cutibacterium acnes* is particularly notable for driving inflammation under oxidative stress, potentially inducing mutagenesis or epigenetic reprogramming that facilitates tumor development (27, 56, 57).

However, comparative analyses across different cohorts have identified inconsistent enrichment patterns—for example, Lachnospira has been reported as both increased (56) and decreased (44) in PCa, underscoring divergent results that may reflect population differences or methodological variability. Many studies rely on small, geographically homogeneous cohorts, raising concerns about the generalizability of reported microbial signatures. Geographic, dietary, and ethnic differences can profoundly influence microbiome composition, making it difficult to discern universal cancer-associated taxa from population-specific patterns. Furthermore, the predominance of 16S rRNA gene sequencing, although invaluable for initial profiling, restricts taxonomic resolution and offers limited insight into functional potential (58–60). In addition, 16S-based approaches are unable to detect gene-level microbial functions and may mis-classify closely related taxa, whereas shotgun metagenomics provides higher-resolution insights but is rarely used due to higher costs and stringent data requirements. Shotgun metagenomics, metatranscriptomics, and integrated host-microbe multi-omics are increasingly regarded as essential to move beyond associative evidence and uncover causative mechanisms in PCa. However, inconsistencies in sampling protocols, such as differences between transrectal versus transperineal biopsy routes and inadvertent contamination from urethral flora, together with the absence of standardized bioinformatic pipelines, continue to hinder robust cross-study comparisons (61–63).

In summary, current evidence supports a biologically plausible link between the microbiome and PCa pathogenesis, with both local prostatic communities and distant niches such as the gut and urinary tract contributing to the disease process. To move from correlation to causation, future studies must integrate multi-centre cohorts and functional multi-omics analyses capable of identifying true microbial drivers of tumor initiation and progression. Importantly, this mechanistic understanding sets the stage for the next section, which explores how inter-organ microbial interactions, particularly between the prostatic, urinary and gut microbiomes, actively shape tumor progression.

### Factors contributing to prostatic dysbiosis

Dysbiosis refers to the disruption of a healthy microbial balance, resulting in alterations in community structure, diversity, and functional capacity (64). In the context of the prostate, the precise etiology of dysbiosis remains incompletely elucidated, partly because sampling the organ for microbial analysis is inherently invasive, limiting large-scale studies and longitudinal monitoring. Nevertheless, current evidence suggests that microbial translocation from both the gut and the genitourinary tract constitutes a plausible source of prostatic colonization, particularly in states of impaired epithelial barrier function or immune modulation (65). Age-related shifts in microbial diversity are a critical factor, as declines in species richness and evenness in both the gut and distal urethra have been correlated with compositional changes in the prostatic microenvironment, potentially altering immune surveillance and metabolic interactions (28, 66). Diet emerges as another potent modulator, high-fat dietary patterns have been shown to enrich *Firmicutes* abundance, a change associated with systemic low-grade inflammation, metabolic dysregulation, and possible facilitation of PCa progression through immune and hormonal pathways (35, 67). Hormonal modulation further influences the microbial landscape. ADT, a cornerstone in advanced PCa management, not only induces profound systemic hormonal shifts but also significantly reduces both *α*- and *β*-diversity in the gut microbiota. This reduction may exacerbate dysbiosis, impair gut-derived immune regulation, and potentially influence tumor biology through altered metabolite production and immune signaling (68, 69). Infectious history represents an additional layer of complexity. Prior bacterial infections, such as *Escherichia coli*-induced prostatitis, can disrupt epithelial integrity, trigger chronic inflammatory cascades, and create a microenvironment conducive to persistent microbial shifts (70). Chronic inflammation, in turn, may facilitate selective enrichment of pathobionts and depletion of protective taxa, reinforcing a cycle of dysbiosis and tissue injury. Moreover, antibiotic exposure highlights the fragility of the prostatic microbial ecosystem. Both short and long-term antibiotic use can induce broad-spectrum alterations in the intestinal and urethral microbiota, with downstream consequences for the prostate. Such perturbations may deplete beneficial commensals, enable opportunistic colonization, and indirectly remodel the prostate’s microbial community through immune and metabolic pathways (71). Taken together, these findings underscore that prostatic dysbiosis is the product of multifactorial influences, ranging from aging and diet to hormonal therapy, infection, and antimicrobial interventions, interacting within a delicate and interconnected host-microbe network.

## Interactions between prostatic, urinary, and gut microbiomes in tumor progression

**Figure 1:**
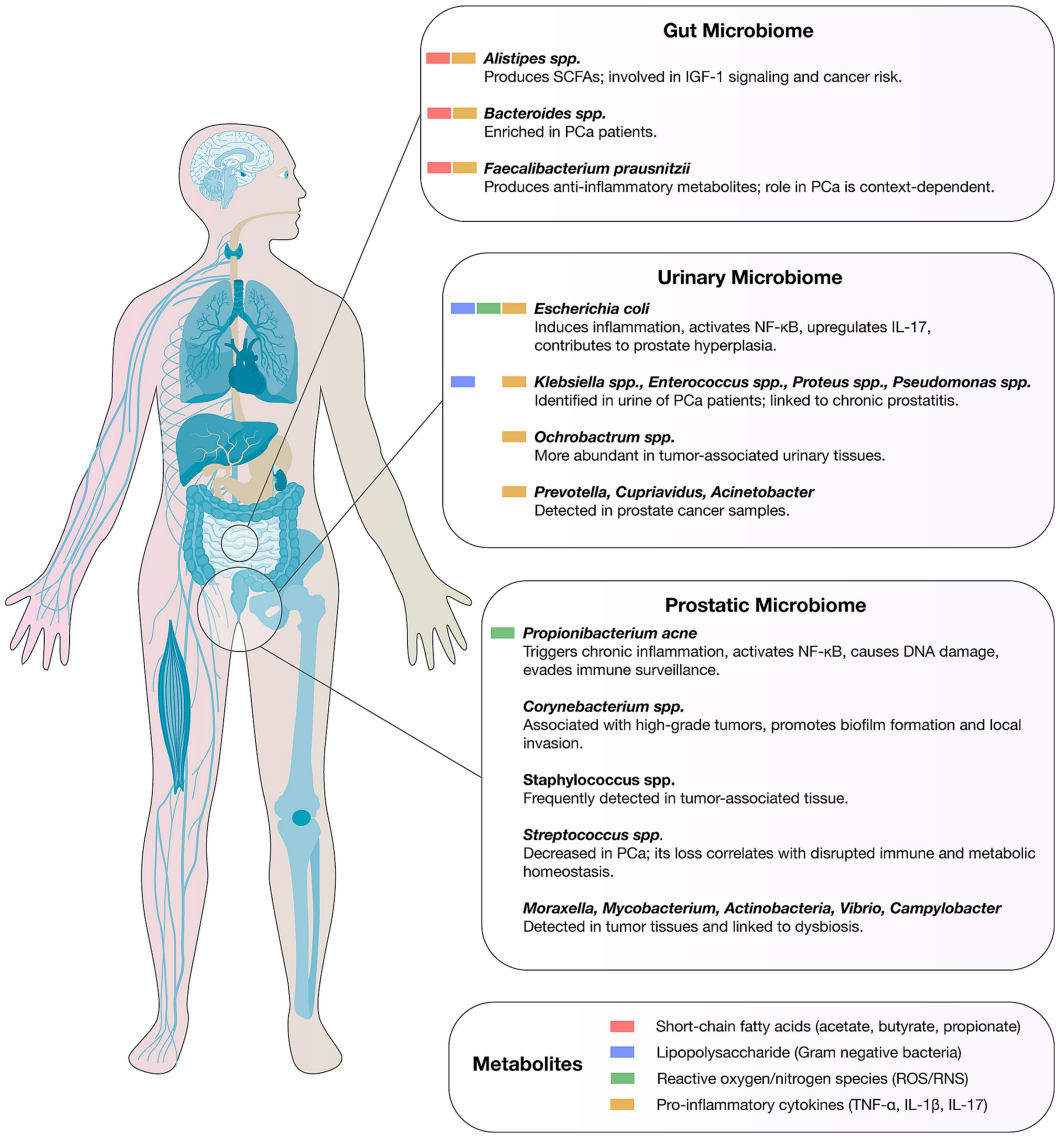
Microbial signatures of the gut, urinary, and prostatic microbiomes in prostate cancer. The figure depicts representative bacterial taxa and key microbial metabolites for each cancer type.

Emerging evidence indicates that the prostatic, urinary, and gut microbiomes form an interconnected axis that can shape PCa initiation, progression, and therapeutic response through overlapping immunological, metabolic, and inflammatory pathways (Figure 1). Recent 16S rRNA sequencing studies in urine have detected genera such as *Blautia* and *Ruminococcus*, further supporting the concept of a shared microbial reservoir between urinary and prostatic niches (72, 73). Metagenomic, culture-based, and transcriptomic profiling of urine, prostatic fluid, and prostate tissue reveal distinct microbial signatures linked to tumor aggressiveness. For example, Hurst et al. identified anaerobic genera, including novel *Porphyromonas*, Var*ibaculum*, *Peptoniphilus*, and *Fenollaria* species, whose presence in urine sediment, urinary extracellular vesicles, and cancer tissue correlated with higher D’Amico risk groups and metastatic progression, conferring a meta-analysis hazard ratio of 2.60 for disease progression (74). These organisms may traffic between the urinary tract and prostate via ascending infection or vesicle-mediated transfer, as suggested by the detection of identical taxa across these niches, with potential prognostic utility. Chronic inflammation emerges as a central mechanistic bridge in this tri-microbiome axis: histological prostatitis and proliferative inflammatory atrophy (PIA) are enriched in immune-infiltrated zones of the peripheral prostate, where most cancers originate, and are often associated with bacterial colonization (43). Persistent infection by *Cutibacterium acnes*, frequently isolated from prostate tissue, urine, and prostatic fluid, elicits cytokine-driven inflammation via NF-κB activation, oxidative stress, and epigenetic alterations, thereby promoting neoplastic transformation (72, 75). Importantly, the predominance of non-cutaneous *C. acnes* genotypes in PCa tissue suggests strain-specific tropism and pathogenicity.

Beyond local interactions, the gut microbiome exerts systemic influence on the prostate through metabolite trafficking, endocrine modulation, and immune crosstalk. In PCa patients, fecal microbiomes enriched for *Bacteroides* and *Streptococcus* display altered metabolic pathway profiles, notably folate and arginine biosynthesism (47), which could impact DNA synthesis and nitric oxide signaling within the tumor microenvironment. Moreover, fecal SCFAs such as butyrate can be taken up by prostatic tissues, suggesting a direct metabolite-mediated axis with potential endocrine relevance. Experimental evidence shows that gut dysbiosis induced by antibiotics enriches *Proteobacteria*, increases gut permeability, and elevates intratumoral LPS levels, thereby activating the NF-κB–IL6–STAT3 axis to enhance proliferation and docetaxel resistance (76). Importantly, fecal microbiota transplantation (FMT) from dysbiotic donors into murine PCa models reproduces this pro-tumorigenic phenotype, demonstrating colonization-dependent transmissibility of oncogenic microbial functions. FMT experiments confirmed that this pro-tumorigenic phenotype can be transferred, underscoring the functional relevance of gut-derived molecules reaching the prostate via systemic circulation. Likewise, gut microbiome alterations in high-tumor-burden PCa patients, when transplanted into mice, accelerated tumor growth; metabolic pathway analysis revealed modulation of long-chain fatty acid metabolism, with omega-3 LCFA supplementation reversing tumor-promoting effects, reducing *Ruminococcaceae* abundance, and lowering fecal butyrate levels in patients (16). These findings illustrate bidirectional metabolite trafficking, in which dietary or microbial metabolites generated in the gut exert endocrine and paracrine effects on the prostate.

The urinary microbiome represents both a conduit and a reflection of prostatic microbial ecology, with high-PSA PCa patients showing reduced prostatic fluid microbial diversity and a differential enrichment of *Enterobacter*, *Lactococcus*, and *Streptococcus* compared to controls (77). These genera, present in both urinary and gut compartments, may signify translocation events through hematogenous or lymphatic routes, or via contiguous mucosal surfaces. The urinary microbiome also holds potential as a non-invasive biomarker for PCa detection and risk stratification, with infection-inflammation cycles originating from urinary tract colonizers capable of remodeling the prostatic microenvironment (24). Furthermore, ADT has been shown to alter gut microbiota composition, reducing diversity and shifting metabolite profiles toward increased lactate and butyrate (78), which may feed back into tumor biology by modulating systemic metabolic and immune tone.

Mechanistic parallels between the three microbiomes are evident in their capacity to sustain chronic inflammation, remodel immune infiltration, and alter local metabolite landscapes. Prostatic immune cell populations, including cytotoxic CD8^+^ T cells and regulatory T cells, are sensitive to microbial and metabolite cues from both local and distal niches (43). Gut-SCFAs, while generally anti-inflammatory in healthy states, may under certain dysbiotic conditions promote tumor growth via IGF-1 pathway activation or influence androgen metabolism (16, 76). Conversely, urinary tract colonization by anaerobes capable of producing volatile fatty acids, amines, and reactive oxygen species (ROS) can directly damage epithelial DNA, foster PIA, and facilitate oncogenic mutations (72, 74). The clinical relevance of this inter-niche trafficking is underscored by observations that microbial and metabolite signatures in gut and urine samples can outperform PSA in predicting metastatic status (76) and may serve as therapeutic targets via dietary, probiotic, or FMT-based interventions (16, 24).

Collectively, these findings support a model in which the prostatic, urinary, and gut microbiomes form a functional axis wherein bacteria, their metabolites, and immune-modulating molecules can traverse anatomical and physiological barriers to influence tumor progression. The gut supplies endocrine-active metabolites and inflammatory mediators that reach the prostate through circulation, while urinary and prostatic niches exchange microbes and molecular signals through direct anatomical continuity. Such cross-compartmental trafficking is clinically relevant not only for biomarker development but also for therapeutic modulation: strategies aimed at restoring microbial balance or blocking deleterious metabolite flux, such as targeted antimicrobials against pathogenic *C. acnes* genotypes, dietary LCFA modulation, or SCFA pathway reprogramming, hold promise for mitigating PCa progression and improving treatment responsiveness. Future studies should integrate multi-omics approaches and longitudinal sampling to clarify causal links and exploit inter-microbiome dynamics for precision oncology, integrating microbiome profiling across all three compartments to account for the full spectrum of microbial influences on prostate tumor biology (24, 72, 74).

## Integrative mechanistic framework of the gut-urinary-prostate axis in prostate cancer

The emerging concept of the gut-urinary-prostate axis underscores a hierarchically organized microbial network in which metabolites, immune mediators, and microbial molecules act as shared effectors across compartments. Mechanistically, this axis operates through three primary routes: metabolic circulation, immune cross-priming, and direct microbial translocation.

### Metabolic circulation

Gut-derived metabolites, including SCFAs, secondary bile acids, and indole derivatives, enter systemic circulation and accumulate in prostatic tissue, where they modulate epithelial proliferation, AR signaling, and immune tone. For instance, SCFAs such as butyrate and propionate can epigenetically remodel prostate epithelial cells by inhibiting histone deacetylases, while dysbiotic enrichment of Ruminococcaceae and Bacteroides enhances IGF-1/PI3K/AKT signaling, promoting tumor cell survival. Similarly, bile acid–producing taxa drives oxidative stress and DNA methylation changes that support tumor initiation (16).

### Immune cross-priming

Circulating bacterial products (e.g., lipopolysaccharides, peptidoglycans) engage pattern-recognition receptors (TLR4, NOD1/2) in both urinary and prostatic epithelia, leading to chronic NF-κB and JAK/STAT activation, cytokine release, and infiltration of M2-polarized macrophages. This systemic immune imprinting links distal gut dysbiosis to local inflammation in the prostate. Experimental models show that antibiotic-induced depletion of commensals elevates intratumoral IL-6 and STAT3 signaling, thereby enhancing docetaxel resistance (76).

### Microbial translocation and vesicle-mediated trafficking

Whole bacteria or bacterial extracellular vesicles can translocate from the gut to the urinary tract via hematogenous or lymphatic routes. Identical bacterial genotypes, such as *Cutibacterium acnes*, *Porphyromonas*, and *Ruminococcus*, have been isolated from gut, urine, and prostate samples, suggesting bidirectional migration and ecological seeding of the prostatic niche. Vesicle-mediated transfer of microbial RNA, metabolites, and quorum-sensing molecules across these compartments may further reinforce oncogenic signaling networks (72, 74).

These interconnected mechanisms generate a feed-forward loop wherein gut dysbiosis modulates systemic inflammation and hormone metabolism, urinary dysbiosis amplifies local cytokine and oxidative stress responses, and the prostate microbiome integrates these signals to promote neoplastic transformation and therapy resistance. Unlike broader microbiome-oncology frameworks, this integrated model delineates organ-specific microbial crosstalk that uniquely underpins prostate carcinogenesis, providing a mechanistic foundation for the proposed gut-urinary-prostate axis.

## Molecular mechanisms involved in the microbiome-prostate cancer relationship

**Figure 2:**
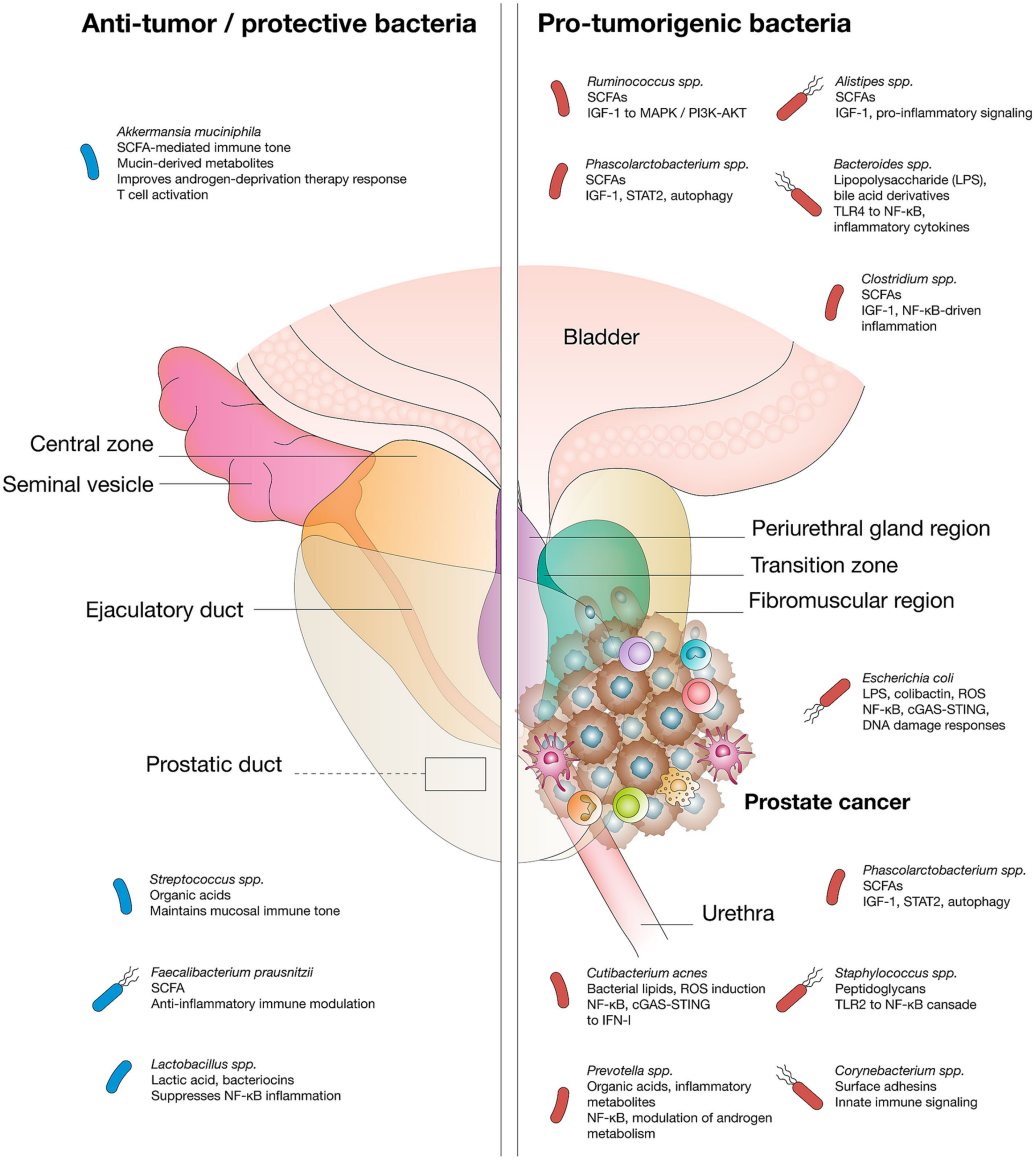
Microbial species mediate pro-tumor or anti-tumor effects via specific metabolites and signaling pathways in prostate cancer.

The prostate is an immunocompetent organ populated by lymphocytes, macrophages, and mast cells, which mount immune defenses against pathogens. With aging, immune cell infiltration increases, predisposing the prostate to chronic inflammation—a recognized driver of neoplastic transformation that fuels both tumor initiation and progression (43, 79, 80). Persistent inflammatory activity, whether triggered by infections (e.g., *Escherichia coli*, *Propionibacterium acnes*) or non-infectious insults such as oxidative stress, results in sustained release of ROS, cytokines, and chemokines, promoting angiogenesis, epithelial-mesenchymal transition, immune evasion, and metastatic potential (81, 82). Dysbiosis in gut, oral, and prostatic microbiota perpetuates these processes by stimulating immune pathways and generating pro-carcinogenic metabolites, particularly SCFAs such as butyrate, acetate, propionate, and isopropionate. Notably, SCFAs can inhibit histone deacetylases, thereby altering chromatin accessibility and transcriptional programs related to cell proliferation and survival (26, 83). In high-grade and castration-resistant PCa, enrichment of SCFA-producing taxa, like *Rikenellaceae*, *Alistipes*, *Lachnospira*, *Ruminococcus*, *Phascolarctobacterium*, correlates with upregulation of IGF-1, which is increased after gut dysbiosis and activates MAPK and PI3K pathways, driving proliferation and survival (18, 33, 84). These inflammatory and metabolic signals converge on canonical oncogenic cascades, notably NF-κB and JAK/STAT, the latter of which can be activated by bacterial peptidoglycan, orchestrating angiogenesis, metabolic rewiring, cellular plasticity, and therapy resistance (85–87). Although indirect, emerging data also suggest that genotoxin-producing bacteria, such as colibactin-producing *Escherichia coli*, may contribute to prostate carcinogenesis by inducing DNA damage and mutational signatures relevant to tumor development (87) (Figure 2).

Crucially, chronic inflammation and microbial dysbiosis intersect with androgen receptor (AR) signaling, a central driver of PCa. Androgens, testosterone and dihydrotestosterone (DHT), regulate prostate growth and oncogenesis, yet their systemic levels can be shaped by gut microbiota via host modulation and direct androgen biosynthesis (88–90). In murine and human studies, specific bacterial taxa have been shown to synthesize androgenic steroids, reactivating AR pathways even under ADT, thereby fostering castration-resistant disease (17, 91). ADT itself can remodel the gut microbiome, enriching androgen-producing bacteria and further entrenching resistance mechanisms (92, 93). Moreover, diet-microbiota-androgen interactions modulate these effects, as seen in castrated mice on high-fat diets exhibiting shifts in the *Firmicutes/Bacteroidetes* ratio and enrichment of *Lactobacillus* species (94).

Beyond these inflammatory, metabolic, and hormonal pathways, recent evidence highlights the critical role of miRNAs as post-transcriptional regulators that are strongly influenced by microbial activity. Dysbiosis-associated metabolites, particularly SCFAs, can modulate the expression of tumor-suppressive miRNAs (e.g., miR-34a and miR-200c), thereby attenuating epithelial-mesenchymal transition and cell proliferation; conversely, proinflammatory microbiota enhance the expression of oncogenic miRNAs such as miR-21 and miR-155, promoting immune evasion and metastatic dissemination (40, 95). Moreover, several microbiome-regulated miRNAs directly modulate androgen receptor signaling and chromatin structure, contributing to resistance to ADT (41, 96). Microbial metabolites also influence DNA methyltransferase and histone acetyltransferase activity, reshaping the transcription of miRNA clusters that are directly implicated in PCa progression and therapeutic resistance (97). Collectively, these findings position the microbiome as a master regulator of epigenetic and miRNA-mediated mechanisms, reinforcing its role as both a mechanistic driver and a promising therapeutic target in PCa.

## Therapeutic strategies based on the modulation of the microbiome in prostate cancer

The human microbiome exerts dual influences on PCa, displaying both pro-tumorigenic and anti-tumorigenic properties through mechanisms such as modulation of immune responses, production of microbial toxins, and secretion of metabolites that trigger oxidative stress, DNA damage, and chronic inflammation (98, 99). These activities shape the tumor microenvironment and interact with systemic therapies, including chemotherapy, hormone therapy, immunotherapy, and radiotherapy, thereby influencing treatment outcomes (100).

A growing body of evidence demonstrates that the microbiome modulates the pharmacodynamics and toxicity of chemotherapeutic agents. Certain bacterial taxa metabolize chemotherapeutic drugs, altering their activity and side effect profiles. Cyclophosphamide, for example, induces villous atrophy in the intestine, enhancing permeability and facilitating bacterial translocation into the systemic circulation. Furthermore, the microbiome has been implicated in mitigating radiation-induced gastrointestinal toxicity, underscoring its relevance for improving the tolerability and efficacy of cancer therapies (101–103).

In immunotherapy, the gut microbiome has emerged as a critical determinant of response, including in PCa. Microbial composition influences the therapeutic efficacy of immune ICIs, particularly anti-CTLA-4 and anti-PD-L1 antibodies (104, 105). Specific commensal species can enhance antitumor immunity, thereby potentiating immunotherapy efficacy (106, 107). Notably, CTLA-4 blockade exhibits greater dependency on a supportive microbiome than PD-L1 inhibition. While CTLA-4 antibodies require favorable microbial communities for optimal activity, PD-L1 inhibitors are less dependent on gut microbiota and do not cause significant intestinal toxicity (108, 109). Importantly, PD-L1 expression is upregulated in PCa cells following infection, promoting immune evasion and tumor progression, further highlighting the centrality of microbiota-immune crosstalk in therapeutic modulation (110–112).

ADT remains the cornerstone of systemic treatment for advanced PCa; however, it exerts profound effects on the gut and prostatic microbiomes that extend beyond endocrine suppression. ADT fosters the expansion of bacterial species capable of converting precursor molecules into active androgens, thereby sustaining tumor growth and promoting therapeutic resistance (34, 68). This underscores a bidirectional interaction wherein gut dysbiosis impairs drug metabolism while ADT itself reshapes microbial ecology.

Recent mechanistic evidence demonstrates that ADT reshapes microbial community structure, decreasing overall *α*-diversity and favoring the outgrowth of commensals with steroidogenic capacity. These bacteria possess genes encoding 17β-hydroxysteroid dehydrogenase and 3β-hydroxysteroid dehydrogenase, which catalyze the conversion of adrenal and dietary precursors, such as dehydroepiandrosterone and androstenedione, into bioactive testosterone and dihydrotestosterone, thereby sustaining intratumoral androgen receptor (AR) signaling even under castration conditions (17). In preclinical murine models, antibiotic-mediated depletion of these steroidogenic taxa restored ADT sensitivity and delayed the onset of castration-resistant prostate cancer (CRPC), demonstrating a causal link between microbial androgen biosynthesis and therapy resistance. Parallel metagenomic analyses in PCa patients undergoing enzalutamide or abiraterone treatment have revealed enrichment of *Ruminococcus*, *Bacteroides*, and *Prevotella* species harboring CYP450 and steroid dehydrogenase genes, corroborating the concept of a “microbial androgen factory” that perpetuates AR activation despite pharmacologic suppression (34, 78).

Mechanistically, ADT-induced dysbiosis contributes to resistance through dual routes: (i) microbial androgen biosynthesis, maintaining AR-dependent transcriptional programs; and (ii) immunometabolic reprogramming, characterized by elevated short-chain fatty acid production (notably butyrate and propionate) that enhances Treg expansion and dampens antitumor immunity. This underscores a bidirectional interaction wherein gut dysbiosis impairs drug metabolism while ADT itself reshapes microbial ecology. Collectively, these effects establish a feedback loop in which endocrine therapy alters the microbiome, and the altered microbiome, in turn, subverts endocrine control. Therapeutic opportunities are emerging from this paradigm. Targeted modulation of gut microbiota, through dietary interventions, probiotics such as *Akkermansia muciniphila* that enhance ADT responsiveness, or selective antibiotic regimens aimed at steroidogenic taxa, could restore hormonal sensitivity and prevent CRPC evolution. Future translational efforts integrating metagenomic, metabolomic, and host transcriptomic profiling will be essential to stratify patients based on microbial steroidogenic capacity and identify those most likely to benefit from combinatorial microbiome–endocrine interventions (16, 78).

The role of antibiotics in this context is complex and paradoxical. Broad-spectrum antibiotics can suppress proinflammatory and SCFA-producing bacteria but at the same time disrupt core microbial networks, diminishing the efficacy of systemic therapies. Fluoroquinolones such as norfloxacin, ciprofloxacin, ofloxacin, and fleroxacin exhibit high prostate tissue penetration (113–115), yet their influence on the prostate microbiota remains incompletely understood. Antibiotics alter microbial diversity and metabolic activity, potentially modifying therapeutic responses (76, 116). Some reports suggest that penicillins, tetracyclines, quinolones, and sulfonamides may reduce PCa risk by attenuating inflammation and modulating microbial composition, although these observations require further validation (117, 118).

Recent advances have turned attention to FMT as a strategy to restore eubiosis and augment therapeutic outcomes in PCa (24). FMT aims to correct dysbiosis by transferring fecal material from healthy donors to patients, thereby restoring microbial diversity and influencing systemic processes such as immune regulation, inflammation, metabolite production, and oncogenic signaling (119, 120). Preclinical evidence from transgenic adenocarcinoma of the mouse prostate (TRAMP) models demonstrates that transplantation of fecal microbiota from PCa patients increases the abundance of SCFA-producing bacteria such as *Ruminococcus*, *Alistipes*, and *Phascolarctobacterium*, which have been linked to tumor-promoting metabolic activity (32, 121). These findings suggest that cancer-derived microbial communities may accelerate tumorigenesis via metabolite-driven and immune-mediated pathways. Conversely, FMT has also been shown to reduce DNA damage and exhibit antineoplastic effects, likely mediated through systemic circulation of microbial metabolites. Dietary factors, particularly high-fat diets, further exacerbate dysbiosis and PCa progression by disrupting metabolite production, an effect that FMT and similar microbiota-based interventions could potentially reverse (46, 122).

In parallel, bacteriophage therapy has emerged as a novel microbiome-modulating approach in light of increasing antibiotic resistance. Phages offer high specificity against pathogenic bacteria, are inherently non-pathogenic to humans, and can be engineered for precision targeting (123). This strategy is being investigated in cases of chronic bacterial prostatitis resistant to antibiotics, with promising preliminary results (124, 125). Beyond their antimicrobial activity, bacteriophages may also influence cancer biology. *In vitro* experiments using androgen-responsive LNCaP prostate adenocarcinoma cells have demonstrated that phages modulate the expression of genes involved in cell proliferation, viability, and androgen receptor signaling (126), suggesting that they may serve as modulators of tumor biology in PCa.

Probiotics and prebiotics represent another line of microbiome-centered therapeutic strategies in PCa. Probiotics, defined as live microorganisms conferring health benefits when administered in adequate amounts, and prebiotics, which are non-digestible substrates that selectively stimulate the growth of beneficial bacteria, both aim to restore homeostasis and counteract dysbiosis (46, 70). These interventions modulate host metabolism, enhance immune responses, and promote the production of beneficial metabolites such as SCFAs (127, 128). In PCa, probiotics have been shown to improve therapeutic responses, reduce adverse events, and lower the risk of postoperative infections (129, 130). By restoring microbial equilibrium, these approaches may provide complementary benefits alongside conventional therapies.

Collectively, these microbiome-based strategies highlight the potential of modulating microbial communities to enhance therapeutic efficacy, reduce toxicity, and delay resistance in PCa. Interventions such as FMT, probiotics, prebiotics, bacteriophage therapy, and selective antibiotics converge on a common goal: to reshape microbial composition and function, strengthen host immunity, and suppress tumor-promoting mechanisms. These insights establish the microbiome as a pivotal therapeutic target in PCa, offering opportunities for innovative interventions that integrate microbial, immune, and metabolic networks into cancer management (100, 131, 132) (Table 1).

**Table 1:** Microbiome-targeted therapeutic strategies in prostate cancer.

Therapeutic strategy	Microbiome interaction	Implications in prostate cancer	Key references
Chemotherapy	Bacteria metabolize drugs, modifying efficacy/toxicity (e.g., cyclophosphamide increases gut permeability, promotes bacterial translocation).	Influences drug pharmacodynamics and toxicity; microbiota can mitigate radiation-induced GI toxicity.	(76)
Immunotherapy	Microbial composition modulates response to ICIs; CTLA-4 more dependent on microbiota than PD-L1. Certain commensals enhance antitumor immunity.	Gut microbiota determine efficacy of immune checkpoint blockade; infections upregulate PD-L1, aiding immune evasion.	(16, 143, 144)
Androgen deprivation therapy	ADT alters gut microbiome, promoting bacteria that convert precursors into active androgens.	Sustains tumor growth, contributes to therapeutic resistance; highlights bidirectional microbiome-ADT relationship.	(16, 17)
Antibiotics	Broad-spectrum antibiotics disrupt microbiota diversity; fluoroquinolones penetrate prostate tissue. Some reduce inflammation and PCa risk.	Dual effect: may reduce infection/inflammation but impair systemic therapy efficacy. Role in PCa risk modulation remains unclear.	(76, 117)
Fecal microbiota transplantation	Restores microbial diversity and eubiosis; modifies SCFA production, immune regulation, inflammation.	Potential to delay PCa progression, reduce DNA damage, and counteract dysbiosis; diet (e.g., high-fat) modulates outcomes.	(16, 144)
Bacteriophage therapy	Targets pathogenic bacteria with high specificity; phages can modulate tumor cell gene expression.	Promising for antibiotic-resistant prostatitis; experimental evidence suggests phages may influence androgen signaling and tumor biology.	(145, 146)
Probiotics and prebiotics	Promote eubiosis, enhance immune responses, stimulate beneficial SCFA production.	Improve therapeutic responses, reduce side effects, lower infection risk, support standard therapies.	(16, 147, 148)

Key therapeutic approaches that modulate or interact with the microbiome in the context of prostate cancer.

## Limitations of current evidence in microbiome prostate cancer research

Despite the increasing number of studies linking gut, urinary-tract and prostatic microbial communities to prostate cancer (PCa), the field remains constrained by several fundamental limitations that must be explicitly addressed to ensure rigorous interpretation and translational relevance.

### Causality versus association

Most published investigations to date are cross-sectional or case–control in design, and thus inherently correlational rather than mechanistic. For example, meta-analysis of seven studies found that gut microbial *α*-diversity was significantly lower in PCa patients than controls, yet the authors concluded that the “limited quality and quantity of selected studies” preclude definitive conclusions about causality (133). Without longitudinal sampling, prediagnostic cohorts or experimental modulation, it remains unclear whether dysbiosis is a cause of tumor initiation/progression, a consequence of malignancy (or its therapy), or a co-incident phenomenon. The recent Mendelian-randomization study suggesting that higher abundance of *Bifidobacterium* may inhibit PCa progression via CD39^+^ Treg mediation is an important advance, but still limited in inferring direct biological pathways in humans. As such, translational claims (e.g., probiotic interventions to prevent PCa) remain premature (70, 134).

### Heterogeneity of findings

A second major limitation arises from inconsistent and sometimes contradictory microbial signatures across studies. While some cohorts identify enrichment of genera such as *Bacteroides*, *Alistipes* or Lachnospiraceae in aggressive PCa, others do not replicate these associations (21). Variations in patient diet, androgen-deprivation therapy status, prior antibiotic exposure, geography, prostate biopsy method and comorbidities likely contribute to this heterogeneity. The lack of reproducible “microbiome signature” for PCa undermines biomarker development and impedes meta-analytical synthesis (135).

### Methodological variability

The landscape of methodology in microbiome-PCa research is highly variable, which diminishes cross-study comparability. Differences include sample source (fecal, urine, prostatic tissue), collection timing (pre- or post-therapy), sequencing strategy (16S rRNA gene amplicon vs. shotgun metagenomics), bioinformatic processing pipelines and contamination control. Reviews repeatedly highlight that many studies rely on 16S sequencing with limited taxonomic and functional resolution, and often lack rigorous designs for microbial causality or mechanistic inference (136). In addition, machine-learning based classification in microbiome studies may suffer from overfitting or inadequate external validation (137). Such methodological heterogeneity reduces the reliability of aggregate conclusions and slows translational progress.

### Population diversity and generalisability

Most studies to date have been conducted in relatively small, ethnically homogeneous cohorts, often in Asia, North America or Europe, with limited inclusion of other populations. The potential influence of host genetics, diet, microbiome baseline composition, healthcare access and therapy patterns on microbial–PCa interactions remains under-explored. Therefore, the reproducibility and generalisability of reported associations to broader global populations are uncertain. Without multi-centre, multi-ethnic sampling and robust cohort sizes, biomarkers or microbiome-based interventions may fail in different demographic settings (138, 139).

### Clinical translation challenges

Even when associations are identified, translation into clinical practice is still nascent. Validated microbial biomarkers for PCa risk stratification, progression or therapy-response prediction are lacking. Interventional studies using probiotics, prebiotics, microbiota transplantation or microbial metabolite modulation are at a very early stage, hampered by uncertainties in optimal strain selection, dosage, treatment duration and safety (70). Moreover, the bidirectional nature of microbiome–therapy interactions (e.g., hormonal therapy altering gut microbiota, microbiota influencing therapy response) complicates clinical implementation. Finally, regulatory, manufacturing and standardization issues of microbiome-based therapeutics remain unresolved (16, 20).

## Analytical and screening approaches for microbiome profiling in prostate cancer

Methodological diversity has greatly influenced the heterogeneity of findings across prostate microbiome studies. Traditional 16S rRNA sequencing remains the most widely applied approach for taxonomic profiling of bacterial communities in gut, urinary, and prostatic samples, yet its limited resolution hampers the detection of strain-level differences and functional gene content. To overcome these constraints, recent investigations have adopted shotgun metagenomics and metatranscriptomics, which provide higher sensitivity and enable simultaneous assessment of microbial composition and metabolic potential. These approaches allow the identification of key functional genes involved in androgen metabolism, inflammation, and immune modulation that 16S-based pipelines cannot capture (61, 63).

Parallel advances in metabolomics (NMR and LC–MS/MS) and metaproteomics have revealed microbial-host metabolic exchanges linked to proinflammatory and androgenic pathways, while host-microbe interactomics integrates microbial metagenomes with host transcriptomes or immune profiling to uncover causal axes between dysbiosis and tumor progression (100). In parallel, *in situ* hybridization, quantitative PCR, and fluorescence microscopy provide spatial resolution of microbial colonization in prostate tissues, complementing sequencing-based datasets (Table 2).

**Table 2:** Analytical methodologies for microbiome profiling in prostate cancer.

Analytical approach	Sample type	Primary readout	Strengths	Limitations	Representative findings in PCa	Key references
16S rRNA sequencing	Stool, urine, prostate biopsy	Taxonomic composition	Low cost, easy bioinformatic processing	Limited functional resolution; prone to contamination	Identification of *Cutibacterium acnes, Streptococcus, Corynebacterium* patterns associated with inflammation	(16, 76, 149)
Shotgun metagenomics	Stool, urine, prostate tissue	Functional genes, microbial pathways	High resolution; detects non-bacterial taxa	Requires deep sequencing; computationally intensive	Discovery of androgen biosynthesis genes in commensals driving ADT resistance	(150, 151)
Metatranscriptomics	Tissue, stool, urine	Active microbial gene expression	Functional and dynamic; links with host pathways	RNA degradation; need for paired host transcriptome	Revealed active expression of inflammatory and steroidogenic genes in prostate lesions	(44)
Metabolomics (NMR, LC–MS/MS)	Serum, urine, feces	Microbial and host metabolites	Functional integration with host metabolism	Metabolite origin sometimes ambiguous	Identified SCFAs and bile acid derivatives influencing AR and NF-κB signaling	(16, 32, 33)
Host-microbe interactomics	Multi-compartmental (gut-urine-prostate)	Integrated gene-metabolite-immune networks	Causal and systems-level insights	High data complexity; need for standardized frameworks	Linked gut dysbiosis to intraprostatic IL-6/STAT3 activation and docetaxel resistance	(17, 76, 152)

Summary of principal analytical approaches used to characterize the microbiome in prostate cancer across different biological compartments. The table compares methodological scope, sample types, analytical outputs, and key findings derived from each platform. While 16S rRNA sequencing remains the most common approach for taxonomic profiling, advanced multi-omics techniques, such as shotgun metagenomics, metatranscriptomics, metabolomics, and host-microbe interactomics, enable functional and mechanistic interpretation of microbial-host interactions underlying androgen metabolism, immune modulation, and therapeutic resistance. Integration of these methods within standardized pipelines is critical to improve reproducibility and translational applicability of microbiome-based discoveries in prostate cancer research.

To ensure reproducibility, standardized pipelines for sample collection (transrectal vs. transperineal biopsy, urine, stool, or seminal fluid) and contamination control are critical, as inconsistent protocols contribute substantially to conflicting results across cohorts (31). Integrating these multi-layered datasets through systems biology and network modeling represents the next frontier toward functional interpretation of the prostate microbiome and its translational application in diagnostics and therapy.

## Conclusions and future perspectives

Accumulating evidence clearly demonstrates that the human microbiome, spanning the gut, prostate, and urinary tract, represents a dynamic and functionally interconnected network that profoundly influences the initiation, progression, and therapeutic responsiveness of PCa. Once considered a peripheral element in urologic oncology, these microbial ecosystems are now recognized as central regulators of host immune surveillance, metabolic homeostasis, and endocrine signaling. Dysbiosis across these compartments has been shown to foster protumorigenic conditions by sustaining chronic inflammation, destabilizing epithelial barriers, and modulating androgen receptor signaling, thereby driving neoplastic transformation and tumor progression (51, 92, 140). Moreover, diverging findings across studies highlight the need to identify convergent microbial patterns that consistently associate with distinct stages of disease. Notably, specific bacterial genera, including *Bacteroides* and *Akkermansia*, have emerged as key mediators of tumor-host crosstalk. These taxa modulate local and systemic cytokine networks and influence the recruitment, differentiation, and functional polarization of immune infiltrates within the tumor microenvironment (65, 70). An important outstanding question is whether specific prostatic microbial signatures are predictive of therapeutic response and can be used to stratify patients prospectively. Beyond their role in carcinogenesis, microbial communities are increasingly implicated in shaping therapeutic outcomes. More recently, microbiota-driven modulation of treatment response has been reported. Distinct microbial configurations appear to potentiate the effects of ADT, whereas other taxa engage in metabolic reprogramming or immunosuppressive signaling pathways that facilitate treatment resistance (68).

These insights have catalyzed a paradigm shift toward microbiome-informed precision oncology. The integration of metagenomic, transcriptomic, metabolomic and host-microbe interactomic approaches is uncovering microbial signatures predictive of disease onset, biological aggressiveness and treatment response (100, 141). Beyond biomarker discovery, microbiome-modulating interventions such as dietary modulation, prebiotics/probiotics and FMT are being explored as strategies to restore eubiosis and enhance host antitumor immunity (102). However, it remains unclear whether specific combinations of microbial taxa and host molecular markers could serve as composite predictors of durable treatment response, representing a second key outstanding question. Importantly, the emergence of precision microbiome modulation offers the potential to mitigate off-target effects associated with conventional therapies while enhancing their efficacy (42). In parallel, bacteriophage therapy, long overlooked, is re-emerging as a precision tool capable of selectively eliminating pathogenic microbial taxa, preserving beneficial commensals, and thereby reducing collateral microbial damage caused by broad-spectrum antibiotics (123).

Despite significant progress, critical challenges persist in unraveling the microbiome’s role in PCa. Establishing causal links between microbial communities and tumor biology will require longitudinal, multi-omic, and mechanistically driven studies (19, 142). Multicenter, well-controlled trials with standardized biospecimen protocols and integrated omics analyses are urgently needed to validate microbial biomarkers and translate them into clinical practice. Similarly, the harmonization of protocols for biospecimen collection, sequencing, and bioinformatic analysis is necessary to enable reproducible and clinically interpretable results across independent cohorts. Translation to the clinic also demands validation in ethnically diverse populations and across different stages of disease to ensure generalizability. A third outstanding question concerns whether modulating the microbiome during therapy could actively reverse resistance mechanisms and improve long-term outcomes.

Taken together, these findings underscore that disentangling the complex and dynamic interplay between microbial ecosystems and tumor biology has the potential to radically transform PCa management. As our understanding of host-microbiome interactions deepens, microbial profiling is poised to become an integral component of clinical practice, enabling improved risk stratification, refined prediction of therapeutic response and the development of microbiota-informed interventions to enhance efficacy and mitigate resistance. The application of systems biology and integrative multi-omics is redefining the microbiome from a passive inhabitant to an active and adaptable component of the tumor ecosystem. In this emerging paradigm, microbial communities are recognized as critical modulators of tumor initiation, immune dynamics and therapeutic outcomes, highlighting their central role in next-generation precision oncology and offering new opportunities to improve the care of patients with PCa.
